# Synchronous precursor B-cell acute lymphoblastic Leukemia and metastatic neuroblastoma in a child: a rare diagnostic challenge

**DOI:** 10.1093/omcr/omag143

**Published:** 2026-09-25

**Authors:** Tala Dakkak, Leyla Mosalmani, Amaal Adnan, Maysaa Alshamandi, Abdullatif Alfarhan, Aliaa alrifai, Roline Zedan, Arwa Yousef Wassouf, Othman Hamdan

**Affiliations:** Faculty of Medicine, Hama University, Hama, Syrian Arab Republic; Department of Pediatrics, Children’s Hospital Damascus, Damascus, Syrian Arab Republic; Department of Pediatrics, Children’s Hospital Damascus, Damascus, Syrian Arab Republic; Department of Pediatrics, Children’s Hospital Damascus, Damascus, Syrian Arab Republic; Faculty of Medicine, Syrian Private University, Damascus, Syrian Arab Republic; Department of Pediatrics, Children’s Hospital Damascus, Damascus, Syrian Arab Republic; Department of Pediatrics, Children’s Hospital Damascus, Damascus, Syrian Arab Republic; Department of Pediatrics, Children’s Hospital Damascus, Damascus, Syrian Arab Republic; Department of Haematology, Children’s University Hospital, Damascus, Syria

**Keywords:** acute lymphoblastic leukemia, neuroblastoma, synchronous malignancy, Pediatric oncology

## Abstract

The synchronous occurrence of hematologic and solid malignancies in children is rare and poses diagnostic challenges. We report a 5-year-old boy with intermittent fever, hepatosplenomegaly, and a large abdominal mass. Initial laboratory investigations revealed severe anemia, and bone marrow flow cytometry demonstrated an abnormal blast population consistent with precursor B-cell acute lymphoblastic leukemia. Abdominal imaging revealed a heterogeneous right adrenal mass with internal calcifications suggestive of neuroblastoma. Due to the discrepancy between hematologic findings and radiologic features, a bone marrow biopsy showed small round blue cells with rosette-like structures. Immunohistochemistry, elevated neuron-specific enolase and urinary vanillylmandelic acid supported metastatic neuroblastoma. Bone scintigraphy demonstrated uptake in lumbar vertebrae.

The patient was diagnosed with precursor B-cell acute lymphoblastic leukemia and stage IV metastatic neuroblastoma.

This case highlights the importance of considering synchronous malignancies when clinical and radiologic findings are discordant, as early recognition is essential for accurate diagnosis and optimal management.

## Introduction

Acute lymphoblastic leukemia (ALL) is the most common pediatric malignancy in the United States, accounting for 20% of cancers in individuals under 20 years [1]. In contrast, neuroblastoma (NB) is the most common extracranial solid tumor in children, originating from neural crest progenitor cells along the sympathetic nervous system, most frequently in the adrenal glands [2]. The synchronous occurrence of ALL and NB is extremely rare [3]. The evaluation of patients with multiple synchronous neoplasms relies on whole-body imaging techniques and molecular diagnostic tools, playing a crucial role in detecting occult lesions and guiding accurate diagnosis and staging [4]. Here, we report a child presenting with features suggestive of ALL who was ultimately found to have coexisting NB, representing the first reported case from Syria.

## Case report

A previously healthy 5-year-old boy presented to the pediatric hospital in Damascus with a six-week history of intermittent fever. There was no relevant family history of malignancy. On clinical examination, hepatomegaly was noted. On physical examination, posterior cervical lymphadenopathy measuring approximately 1 × 3 × 2.5 cm was noted. On abdominal examination, a large abdominal mass was palpated in the right iliac fossa (about 12 × 10 cm), crossing the midline with poorly defined borders. It was not clinically separable from the liver. The spleen was palpable 5 cm below the left costal margin. Initial laboratory investigations revealed severe anemia (Table 1).

**Table 1:** Initial laboratory blood test.

Test	Value	Unit
Haemoglobin	7	g/dl
WBC	4900	mm^3^
Neutrophils	75	%
Lymphocytes	19	%
Platelet count	170 000	mm^3^
ESR	140	mm/h
Urea	11	mg/dl
Creatinine	0.28	mg/dl
GPT	10	U/l
Uric acid	3	mg/dl
LDH	403	U/l
NSE	298	ng/ml
VMA	196	mg/24 h

WBC: White blood cell, ESR: Erythrocyte Sedimentation Rate, GPT: Glutamic-Pyruvic Transaminase, LDH: Lactate Dehydrogenase, NSE: Neuron-specific enolase, VMA: Vanillylmandelic acid

Flow cytometry analysis of the bone marrow revealed an abnormal blast population accounting for approximately 90% of bone marrow cellularity, characterized by expression of B-cell markers including CD19 (82%), CD10 (86%), HLA-DR (97%), CD34 (40%), and partial expression of TdT (15%). The blasts were negative for myeloid and T-cell markers, supporting the diagnosis of precursor B-cell ALL.

**Figure 1:**
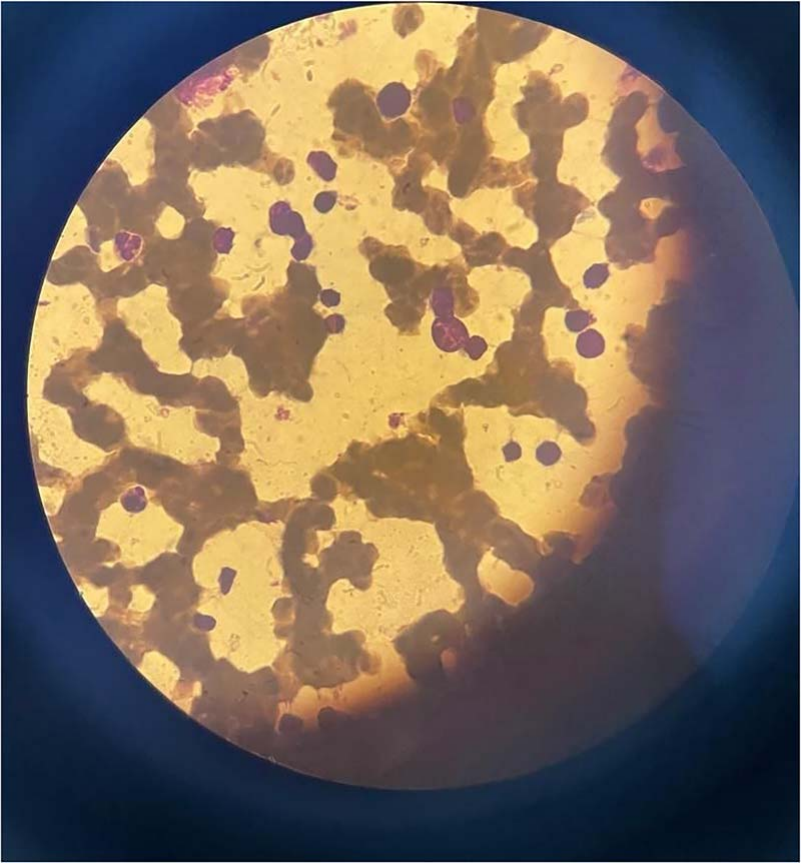
Histopathological section of the bone marrow biopsy showing infiltration by small round blue cells with rosette-like structures characteristic of neuroblastic tumors (H&E stain).

**Figure 2:**
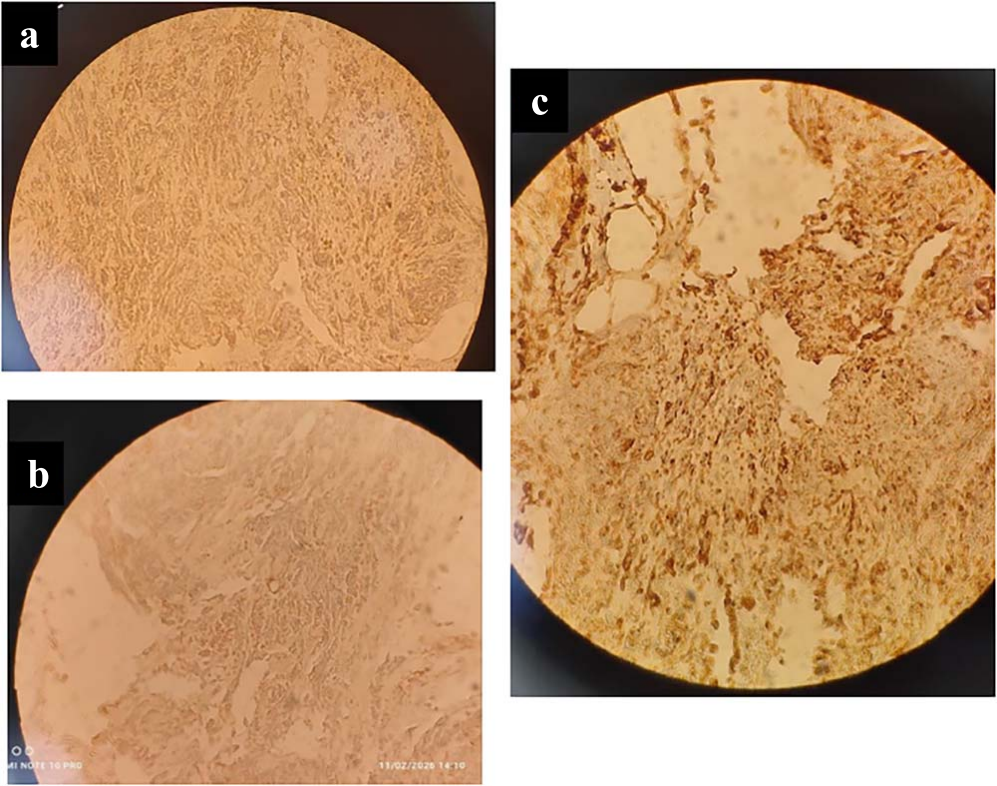
A_C: Immunohistochemical staining of the tumor cells demonstrated positivity for neuroendocrine markers: (a) chromogranin, (b) neuron-specific enolase (NSE), and (c) vimentin.

As part of the diagnostic workup, radiological imaging was obtained, including abdominal ultrasound followed by contrast-enhanced abdominal CT, which revealed a heterogeneous, mixed-density soft-tissue mass containing internal calcifications, arising from the right adrenal region and measuring approximately 4 × 11 × 7 cm. Additional mediastinal lymphadenopathy was noted, raising suspicion for metastatic disease. Due to the inconsistency between the haematologic and radiologic findings, a bone marrow biopsy was performed. Histopathological examination revealed infiltration by small round blue cells, some arranged in rosette-like structures, raising suspicion for a neuroblastic tumor (Fig. 1). Immunohistochemical analysis showed tumor cells positive for chromogranin, neuron-specific enolase (NSE), and vimentin, findings consistent with neuroblastoma (Fig. 2a-c). Laboratory evaluation showed markedly elevated serum NSE levels (298 ng/ml), which supported neuroblastoma, and urinary vanillylmandelic acid (VMA) was also elevated (196 mg/24 h). Bone scintigraphy using technetium-99 m demonstrated increased uptake in the lumbar vertebrae (L4-L5), consistent with skeletal involvement.

In addition, a chest radiograph revealed pleural effusion. Diagnostic thoracentesis revealed a yellowish haemorrhagic pleural effusion, and cytological analysis of the pleural fluid demonstrated malignant cells, confirming pleural metastasis. These findings were consistent with stage IV metastatic neuroblastoma.

Treatment with the SY. Jude Total XVI protocol was initiated, and the patient was referred for multidisciplinary oncologic management and follow-up. Follow-up CT, bone marrow and biopsy examination after the fourth treatment cycle showed no residual malignant infiltration, indicating a favorable response to therapy.

## Discussion

To the best of our knowledge, this is the first reported case in Syria of concurrent NB and pre-B ALL, highlighting a rare and diagnostically challenging presentation. In our patient, the presence of intermittent fever, pallor, fatigue, and lower limb pain is consistent with the typical clinical features of ALL. Additionally, hepatomegaly, splenomegaly, and cervical lymphadenopathy further support the pattern of extramedullary involvement described in the literature. In our case, a large abdominal mass measuring approximately 12 × 10 cm was detected in the right iliac fossa, extending across the midline and exhibiting poorly defined borders, consistent with previously reported features of abdominal NB. The coexistence of leukemia-like systemic manifestations and a large abdominal mass in this patient highlights the significant clinical overlap between ALL and NB, which may lead to diagnostic confusion, particularly in cases with bone marrow involvement. Unlike many other malignancies, the diagnosis of ALL cannot rely solely on morphology [5]. Morphological evaluation identifies blast cells but cannot reliably determine lineage using immunophenotyping, most commonly by flow cytometry. Given the high suspicion of a hematologic malignancy, bone marrow aspiration was performed. Some tumor cells were arranged in rosette-like structures, raising suspicion of neuroblastic differentiation. Bone marrow infiltration by metastatic neuroblastoma may occasionally mimic hematologic malignancies, creating a potential diagnostic challenge [6]. Blast cells accounted for more than 90% of bone marrow cellularity, consistent with acute leukemia. However, the presence of rosette-like structures raised suspicion of possible neuroblastic differentiation. Immunophenotyping by flow cytometry performed on the bone marrow sample confirmed the diagnosis of precursor B-cell ALL. According to the International Neuroblastoma Staging System (INSS), neuroblastoma can be diagnosed based on typical histopathologic findings, elevated urinary catecholamine metabolites, or bone marrow involvement. In our case, neuroblastoma diagnosis is typically established by histopathology and supported by elevated catecholamine metabolites or bone scintigraphy uptake. In our case, the presence of a large abdominal mass with calcifications on imaging was highly suggestive of neuroblastoma. Due to the discrepancy between the hematological findings and the radiological features suggestive of neuroblastoma, a bone marrow biopsy was performed and demonstrated histological features consistent with metastatic neuroblastoma, leading to the diagnosis of stage IV disease. Bone marrow involvement is a common feature of advanced neuroblastoma and represents a defining characteristic of stage IV disease according to the INSS classification [2]. The synchronous presence of precursor B-cell ALL and NB is extremely rare and may create significant diagnostic confusion as metastatic NB can mimic hematologic malignancy. Leukemia has more commonly been reported as a therapy-related secondary malignancy following treatment for neuroblastoma [3]. Therefore, our case may represent the second documented case worldwide and the first reported case from Syria.

However, the coexistence of metastatic neuroblastoma posed a significant diagnostic and therapeutic challenge.

In conclusion, Early recognition of synchronous malignancies is crucial for accurate diagnosis and optimal therapeutic planning, as illustrated by our case highlights the importance of considering a second malignancy when clinical and radiological findings are discordant.
